# COVID-19-Associated Acute Myocarditis: Risk Factors, Clinical Outcomes, and Implications for Early Detection and Management

**DOI:** 10.7759/cureus.44617

**Published:** 2023-09-03

**Authors:** Shiwani Kamath, Mohamad-Hamood T Gomah, Gauthier Stepman, Peter DiMartino, Itioye Adetula

**Affiliations:** 1 Internal Medicine, Medical Center of Trinity, Trinity, USA; 2 Internal Medicine, Hospital Corporation of America (HCA) Florida Trinity Hospital, Trinity, USA; 3 Cardiology, Hospital Corporation of America (HCA) Florida Trinity Hospital, Trinity, USA

**Keywords:** covid-19 and myocarditis, covid-19, covid-19-induced myocarditis, coronavirus disease 2019 (covid-19), myocarditis

## Abstract

Background

The severe acute respiratory syndrome coronavirus 2 (SARS-CoV-2) is a novel coronavirus causing acute respiratory distress with multisystem complications, including cardiac complications. Acute myocarditis is one possible complication of coronavirus disease 2019 (COVID-19). Previous studies revealed that mortality from COVID-19 was higher in patients with cardiac complications.

Objectives

We aim to identify if patients with COVID-19 develop myocarditis and if this condition is associated with an increased incidence of ventilatory support and mortality. We also aim to identify if preexisting cardiac conditions are associated with an increased incidence of ventilatory support and mortality in those who developed COVID-19.

Methods

This is a multicenter, retrospective study including patients aged 18 years and older. Statistical analysis was performed to compare the incidence of in-hospital mortality and ventilatory support in COVID-19-positive patients with and without myocarditis. In this study, we defined myocarditis using elevated troponin-T (TnT) and brain natriuretic peptide (BNP) levels as proxy.

Results

A total of 8,162 patients with a positive COVID-19 polymerase chain reaction (PCR) test were identified. Of those, 1,643 (20.1%) were found to have new-onset acute myocarditis. The risk of ventilation and mortality in these patients was significantly elevated (p<0.001) compared to patients without acute myocarditis. Underlying heart failure was associated with increased odds of in-hospital mortality, which was 1.6 times greater when compared to patients without heart failure. The odds of in-hospital mortality were 2.33 times as likely for those who had non-ischemic cardiomyopathy as opposed to those who did not.

Conclusion

Myocarditis is a serious and potentially fatal complication of COVID-19. The results of this study highlight the importance of routine testing of troponin-T and BNP levels to identify those at risk. Furthermore, underlying heart conditions are associated with a worse outcome, and those patients should be watched closely.

## Introduction

The first cases of severe acute respiratory syndrome coronavirus 2 (SARS-CoV-2) were identified in Wuhan, China, in December 2019. Presumed to originate from the Huanan Seafood Market in South China, the virus spread rapidly and resulted in a disastrous global pandemic [1]. Patients with severe coronavirus disease 2019 (COVID-19) typically develop acute respiratory distress with multisystem complications, leading to a poor prognosis [1]. Cardiovascular implications have largely been unstudied, especially in the United States, as the total count reached 640,014 by April 2020 [2]. Initial studies of 41 hospitalized patients in Wuhan, China, in January 2020 showed that 12% of COVID-19 patients had developed acute myocarditis as evidenced by increased troponin [3]. A more recent study from Wuhan of 138 hospitalized patients documented that 16.7% had arrhythmias and 7.2% had acute cardiac injury. In addition, patients with hypertension and cardiovascular disease were more likely to require intensive care unit (ICU) level care [4]. In another study of 187 participants from Wuhan, 27.8% had a significant increase in cardiac troponin-T (TnT) and brain natriuretic peptide (BNP) levels, demonstrating myocardial injury. Patients with elevated troponin levels were found to have a 50.7% higher mortality rate [5]. The goal of this study was to examine cardiac outcomes in patients with COVID-19 in the United States and assess increased mortality in the setting of known cardiovascular comorbidities or elevated cardiac biomarkers.

## Materials and methods

Study design and setting

This multicenter, retrospective study was performed in the Hospital Corporation of America (HCA) enterprise-wide database of all geographic locations in the United States.

Patients

Study selection criteria included patients 18 years and above who were diagnosed with COVID-19 via polymerase chain reaction (PCR) or antigen testing.

Data collection

The electronic medical records of patients from January 1, 2020, to May 14, 2020, were reviewed by a team of physicians. Abstracted patient data included demographics, medical history, laboratory examinations, comorbidities, complications, treatment measures (such as intubation), and outcomes.

Outcomes

Patients were considered to have acute myocardial injury if they had a laboratory value of TnT or BNP that was above the upper reference limit. Malignant arrhythmia was defined as rapid ventricular tachycardia lasting more than 30 seconds, including hemodynamic instability and/or ventricular fibrillation. The end point was the incidence of COVID-19-associated death.

Statistical analysis

Continuous variables were described as means and standard deviation (SD) or as median and range values. Categorical variables were expressed as counts and percentages. Chi-squared test was used to compare variables between COVID-19 with acute myocarditis and COVID-19 without acute myocarditis groups for categorical data. In-hospital mortality and respiratory support were calculated and adjusted for patient demographics and comorbidities through multivariate analysis. All statistical analyses were performed using Statistical Analysis System (SAS) software (version 9.4), with p<0.05 considered as statistically significant.

## Results

Demographics and clinical characteristics on admission

Data was collected on 8,162 patients with positive COVID-19 PCR or positive antigen results from January 1, 2020, to May 14, 2020, out of which 4,087 were males and 4,075 were females. The median age was 59 years, ranging from 18 to 100 years; the average age of patients was 57.8 years (Table 1). Through self-identified data, 3,920 (48%) patients were white, 2,149 (26.3%) were black, and 2,093 (25.64%) were of other races. Of common preexisting conditions, 902 (35.6%) had hypertension, 1,008 (12.4%) had systolic and/or diastolic heart failure, 1,069 (13.1%) had chronic ischemic heart disease, and 181 (2.2%) had non-ischemic cardiomyopathy. Demographics are presented in Table 1.

**Table 1:** Demographics of patients with COVID-19 Total COVID-19 patient population: N=8,162 COVID-19: coronavirus disease 2019

Demographics		Mean (standard deviation)/number (%)
Age (years)		57.8 (18.8)
Sex	Males	4,087 (50.1%)
	Females	4,075 (49.93%)
Race	Caucasian	3,920 (48%)
	African American	2,149 (26.3%)
	Others	2,093 (25.64%)

Laboratory findings

Of 8,162 patients, 929 (11.44%) had an elevated BNP and 1,056 (12.94%) had an elevated TnT.

Complications by myocarditis

Per our diagnostic criteria, 1,643 (20.1%) COVID-19 patients developed new-onset acute myocarditis. Of COVID-19 patients with acute myocarditis, 37.9% required respiratory support via ventilation during their hospital stay compared to 9% of COVID-19 patients who did not develop acute myocarditis (p<0.0001). Of COVID-19 patients with acute myocarditis, 29.8% experienced in-hospital mortality compared to 5.8% of COVID-19 patients without acute myocarditis (p<0.0001). The chi-squared test results are presented in Table 2.

**Table 2:** Chi-squared tests for outcomes in COVID-19 patients with myocarditis Chi-squared test: p-value below alpha of 0.05 is considered significant. COVID-19: coronavirus disease 2019

Outcome variable	COVID-19 with myocarditis	COVID-19 without myocarditis	Tests for significance
In-hospital mortality	490 (29.8%)	378 (5.8%)	p<0.0001
Respiratory support	623 (37.9%)	587 (9%)	p<0.0001

Complications by preexisting comorbidities

The risk of ventilation requirement was 1.2 times as likely for those who had hypertension versus the normotensive population. The risk of ventilation was 2.3 times as likely for those who had heart failure (systolic with reduced ejection fraction of less than 55% and/or diastolic) as opposed to those without heart failure. The risk of ventilation was 1.584 times as likely for those who had cardiomyopathy as opposed to those who did not. The odds ratios controlled for differences in age, sex, and race that exist within the patient population.

The odds of in-hospital mortality were 0.83 times as likely for those who had hypertension as opposed to normotensive patients, indicating a negative relationship. The odds of in-hospital mortality were 1.6 times as likely for those who had heart failure (systolic failure with reduced ejection fraction of less than 55% and/or diastolic dysfunction) than those without heart failure. The odds of in-hospital mortality were 1.21 times as likely for those who had chronic ischemic heart disease as opposed to those without. The odds of in-hospital mortality were 2.33 times as likely for those who had non-ischemic cardiomyopathy as opposed to those without. These odds ratios are controlled for differences in age, sex, and race that exist within the patient population. The odds ratios and confidence intervals for the main predictor variables are shown in Table 3.

**Table 3:** Odds ratios for comorbidities using logistic regression models for in-hospital mortality and ventilation, controlling for age, sex, and race (N=8,162) N: population size, OR: odds ratio, CI: confidence interval

Comorbidity	In-hospital mortality (N=868)	Ventilation (N=1,210)
Hypertension (OR (95% CI))	0.83 (0.694-0.99) (significant negative relationship)	1.28 (1.10-1.48)
Heart failure (OR (95% CI))	1.64 (1.33-2.02)	2.32 (1.914-2.82)
Cardiomyopathy (OR (95% CI))	2.33 (1.63-3.32)	1.58 (1.130-2.22)
Chronic ischemic heart failure (OR (95% CI))	1.21 (1.01-1.46)	1.16 (0.97-1.37) (not significant)

As an additional note, two (0.02%) patients developed new-onset long QT segments, 17 (0.21%) developed new-onset ventricular fibrillation, and 48 (0.59%) developed new-onset ventricular tachycardia on their electrocardiograms.

## Discussion

Viral infections are a known cause of myocarditis, resulting in a focal to global inflammation of the myocardium [6]. This inflammation can progress to tissue necrosis and subsequent wall dysfunction, raising the risk of sudden cardiac death [6]. Although current literature linking COVID-19 to non-ischemic myocardial injury is insufficient, other forms of coronavirus, such as Middle East respiratory syndrome (MERS) and SARS-CoV, have been found in cardiac tissue of animals [7]. The proposed mechanism of action of COVID-19-induced myocarditis is through direct cell injury and T-lymphocyte-mediated cytotoxicity, further propagated by a cytokine storm [7]. Myocarditis can present as acute-onset heart failure or cardiogenic shock, which in turn causes symptoms that overlap with COVID-19, including dyspnea, cough, and even fevers [7]. These nonspecific symptoms make clinical diagnosis of myocarditis in the setting of COVID-19 difficult.

The diagnosis of myocarditis is made through a combination of laboratory, electrocardiogram (ECG), and imaging studies. Cardiac magnetic resonance imaging (MRI) is the most precise tool for patients with myocarditis, due to its ability to accurately detect inflammation, hyperemia, and the extent of reversible or irreversible cardiac injury. Although it is considered highly specific, the sensitivity of cardiac MRI depends on the protocol of the scan as well as the time points during which the scan was taken and is not generally indicated unless there is diagnostic uncertainty [8]. Although advantageous, due to limited personal protective equipment (PPE) and the goal of limiting exposure, diagnosis via imaging was not done in our study. Prior studies demonstrate that the cardiac biomarker TnT is specific for myocarditis but is 34% sensitive [7]. Since a majority of patients with myocarditis may not have elevated troponin levels, this further establishes that the prevalence of myocarditis may be underestimated in our study. It is important to consider that the number of patients who develop myocarditis may be underestimated further as routine testing of TnT and BNP levels was not typically done on COVID-19-positive patients. Alternatively, it may be considered in future studies to trend TnT levels along with other inflammatory markers to assess any elevation from baseline, especially in patients with preexisting cardiac disease who have chronically elevated biomarkers. Other inflammatory markers, such as lactate, C-reactive protein (CRP), erythrocyte sedimentation rate (ESR), and procalcitonin, are elevated in myocarditis, but these markers were not followed as they are often raised in the presence of infection [7]. ECG changes were found to be vague but included sinus tachycardia and nonspecific ST-segment and T-wave abnormalities, and may be used in conjunction with other studies [9]. In regard to cardiac-specific imaging, the report by Sawalha et al. of 14 cases demonstrated that cardiac MRI was used in 43% of cases, revealing late gadolinium enhancement (LGE) [9]. Newer reports show that COVID-19 can induce hypercoagulability and may result in myocardial infarctions, which can also elevate cardiac biomarkers and cause ST-segment changes [10]. For this reason, patients with elevated troponin may benefit from an ischemic workup.

Through our retrospective study, we found that among 8,162 patients with COVID-19, 929 (11.38%) patients met our diagnostic criteria for myocarditis via elevated TnT or BNP levels. Patients with myocarditis with elevated troponin were more likely to have a poorer prognosis than those with normal troponin levels [7]. In our study, 37.9% of COVID-19 patients with myocarditis required ventilation, while 29.82% of COVID-19 patients with myocarditis expired during their hospital admission. For comparison, a systematic review conducted in 2020 with nine case reports and two additional retrospective studies found similar results to our study, such as COVID-19 myocarditis affecting patients with the same median age over 50 and with near equal incidence between genders [10]. Our study found similar results, with 50.1% of males being affected compared to 49.93% of females. The study also revealed that cardiac markers such as troponin-I, troponin-T, creatine kinase-myocardial band (CK-MB), and BNP were elevated, as well as an increase in white blood cells, CRP, and arterial blood gas analysis, revealing respiratory acidosis [11].

The initial management of fulminant myocarditis is focused on inotropic drugs and vasopressors, followed by mechanical circulatory support with extracorporeal membrane oxygenation (ECMO), ventricular assist device, or intra-aortic balloon pump [7]. The treatment for less severe cases of myocarditis is primarily supportive therapy, but guidelines do not specifically recommend anti-inflammatories. The treatment of COVID-19 myocarditis is evolving as we discover more about the pathogenesis of the infection. In preliminary studies, systemic corticosteroids appear to be the most cardioprotective, with intravenous immunoglobulin (IVIG) and antivirals as secondary options [6]. However, per a Cochrane systematic review, corticosteroids were inadequate in the treatment of viral myocarditis [6]. Per the Randomized Evaluation of COVID-19 Therapy (RECOVERY) trial, dexamethasone was found to decrease mortality in one-third of ventilated patients, but treatment was for other indications. IVIG has a more promising outcome, as a meta-analysis demonstrated that IVIG improved mortality and recovery of left ventricular function in patients with myocarditis [12]. In preliminary studies, remdesivir shortened recovery time in hospitalized patients with COVID-19 but is unclear in its role for myocarditis [13].

It is notable that 67 patients in our study developed new ECG changes. As clinicians previously prescribed antimalarials and macrolides for the treatment of COVID-19, we expect an increased incidence of QTc prolongation. However, our study found only two (0.02%) patients who developed new-onset long QT segments.

In addition, patients with preexisting cardiac conditions were more likely to experience worse outcomes after being hospitalized with COVID-19. Patients with preexisting hypertension, heart failure, and cardiomyopathy were more likely to require ventilation. Those with preexisting heart failure, cardiomyopathy, and chronic ischemic heart failure were more likely to experience in-hospital mortality.

Lastly, although not addressed within the study, we wanted to address the theorized link between COVID-19 vaccinations and myocarditis. It was proposed that because SARS-CoV-2 S glycoproteins have similar structure to cardiac-myosin heavy chain protein, antibodies designed to react against the virus glycoprotein reacted against the cardiac proteins instead, resulting in myocarditis. However, a study comparing several peptides from the SARS-CoV-2 spike to 35 cardiac proteins associated with cardiac autoimmunity found that there were no peptides from the spike that matched any cardiac antigen more than 60% in similarity [14]. Although causal relationships between the administration of vaccines and the development of myocarditis are difficult to ascertain, it has been found that only 0.02% of vaccine recipients in clinical trials for the mRNA-based COVID-19 vaccines developed myocarditis [15]. In a systematic study following 405 million vaccine doses, over 22 different studies found that there was no statistically significant difference in the overall incidence of myocarditis or pericarditis between those with COVID-19 vaccination and those without. It was also found that the risk of myocarditis was higher with mRNA-based vaccines as compared to non-mRNA vaccines as well as the second vaccination dose posing a higher risk for myocarditis than the first-time doses [16]. Overall, the possibility of developing post-COVID-19 vaccination myocarditis is quite rare, and the benefits of receiving the vaccine vastly outweigh the risks.

Study limitations

Firstly, PPE was limited during the initial stages of the pandemic of COVID-19, and as such, we did not use cardiac imaging such as MRI to diagnose myocarditis. Secondly, the underuse of ECG testing other than routine indications may have underestimated arrhythmias.

## Conclusions

Myocarditis is associated with COVID-19 infection and can potentially lead to worse outcomes such as ventilatory support and even death. In the setting of acute respiratory failure from COVID-19, myocarditis is difficult to assess due to overlapping symptoms and may go undiagnosed in COVID-19 patients without routine testing for cardiac biomarkers (TnT and BNP) and cardiac imaging when available. Further studies are warranted to assess the incidence of new-onset cardiac arrhythmias. In conclusion, patients with COVID-19 who develop elevated cardiac biomarkers or have preexisting cardiac conditions carry an increased risk of respiratory failure requiring ventilator support and overall mortality.
